# A case of bacteremia caused by *Dialister micraerophilus* with *Enterocloster clostridioformis* and *Eggerthella lenta* in a patient with pyometra

**DOI:** 10.1186/s12879-024-08999-6

**Published:** 2024-01-24

**Authors:** Hiroki Kitagawa, Kayoko Tadera, Keitaro Omori, Toshihito Nomura, Norifumi Shigemoto, Hiroki Ohge

**Affiliations:** 1 Department of Infectious Diseases, Hiroshima University Hospital, Hiroshima, Japan; 2 Department of Surgery, Graduate School of Biomedical and Health Sciences, Hiroshima University , Hiroshima, Japan; 3 Project Research Center for Nosocomial Infectious Diseases, Hiroshima University, Hiroshima, Japan; 4 Section of Clinical Laboratory, Division of Clinical Support, Hiroshima University Hospital, Hiroshima, Japan; 5 Division of Laboratory Medicine, Hiroshima University Hospital, Hiroshima, Japan; 6 Translational Research Center, Hiroshima University, Hiroshima, Japan

**Keywords:** Anaerobes, Bloodstream infection, Antimicrobial resistance, Gynecological infections, MALDI-TOF MS

## Abstract

**Background:**

Infection by *Dialister micraerophilus*, an obligate anaerobic gram-negative bacillus, has rarely been described, and its clinical characteristics remain unclear.

**Case presentation:**

We report a case of bacteremia caused by *D. micraerophilus*, *Enterocloster clostridioformis*, and *Eggerthella lenta* in a 47-year-old woman, associated with pyometra. *D. micraerophilus* was identified using 16S rRNA gene sequencing and matrix-assisted laser desorption ionization time-of-flight mass spectrometry. *D. micraerophilus* was detected by polymerase chain reaction using *D. micraerophilus-*specific primers and *E. clostridioformis* and *E. lenta* was isolated from the drainage pus sample obtained from the pyometra uterus. The patient achieved a cure after abscess drainage and 2-week antibiotic treatment.

**Conclusions:**

To the best of our knowledge, this is the first report of *D. micraerophilus* bacteremia. *D. micraerophilus* may be associated with gynecological infections. Clinicians should consider both oral and gynecological sites when searching to identify the focus of *D. micraerophilus* infection.

## Background

*Dialister* species are non-fermentative, obligate anaerobic, gram-negative bacillus that are frequently isolated from human clinical samples [1]. Among *Dialister* spp., *D. pneumosintes* is a commensal oral microbe [2], which is mainly associated with oral infections such as gingivitis [3], periodontitis [4], and periapical abscess [5]. *D. pneumosintes* can also cause extra-oral infections such as pneumonia [6], neck and mediastinal abscess [7], sinusitis [8], hepatic abscess [9], and vaginosis [10].

In contrast, *Dialister micraerophilus*, first described in 2005 [11], has been isolated from cutaneous and soft tissue, gynecological, bone, and oral samples [1, 12, 13]. In addition, *D. micraaerophilus* was recently detected from vaginal samples [14, 15]. However, only one infection, a Bartholin’s abscess, has been reported previously as due to *D. microaerophilus* [16].

Herein, we report a case of bacteremia caused by *D. micraerophilus, Enterocloster clostridioformis*, and *Eggerthella lenta* associated with pyometra.

## Case presentation

A 47-year-old Japanese woman was referred to our hospital for suspected endometrial pyometra. This patient, with a medical history of caesarean section 20 years ago, had a 7-day history of genital bleeding and 3-day history of a fever over 38 °C. The initial evaluation at our hospital revealed a body temperature of 38.2 ℃ and no other symptoms suggestive of sepsis, while physical examination revealed lower abdominal pain. The laboratory results were as follows: white blood cell count of 8,670/µL (neutrophils, 88.6%) and C-reactive protein level of 6.02 mg/dL. Transvaginal echocardiography showed an enlarged uterus with accumulation of fluid in the uterine cavity, suggesting pyometra. Drainage of the uterine cavity was performed and purulent fluid was collected, which were submitted for culture. Two sets of blood cultures were also submitted upon admission, and cefmetazole treatment (1 g every 8 h) was empirically started.

Gram-staining of the pus sample showed a polymicrobial pattern. The pus sample was cultured as previously described [17]. Anaerobic conditions were established using an AnaeroPack System anaerobic jar (Mitsubishi Gas Chemical Co., Inc., Tokyo, Japan) equipped with an AnaeroPack (Mitsubishi Gas Chemical Co., Inc.). *Streptococcus gallolyticus* subsp. *gallolyticus, Peptostreptococcus anaerobius, Aerococcus murdochii, Peptoniphilus lacrimalis, E. clostridioformis* (formerly known as *Clostridium clostridioforme*), and *E. lenta*, were identified in the pus sample.

**Fig. 1:**
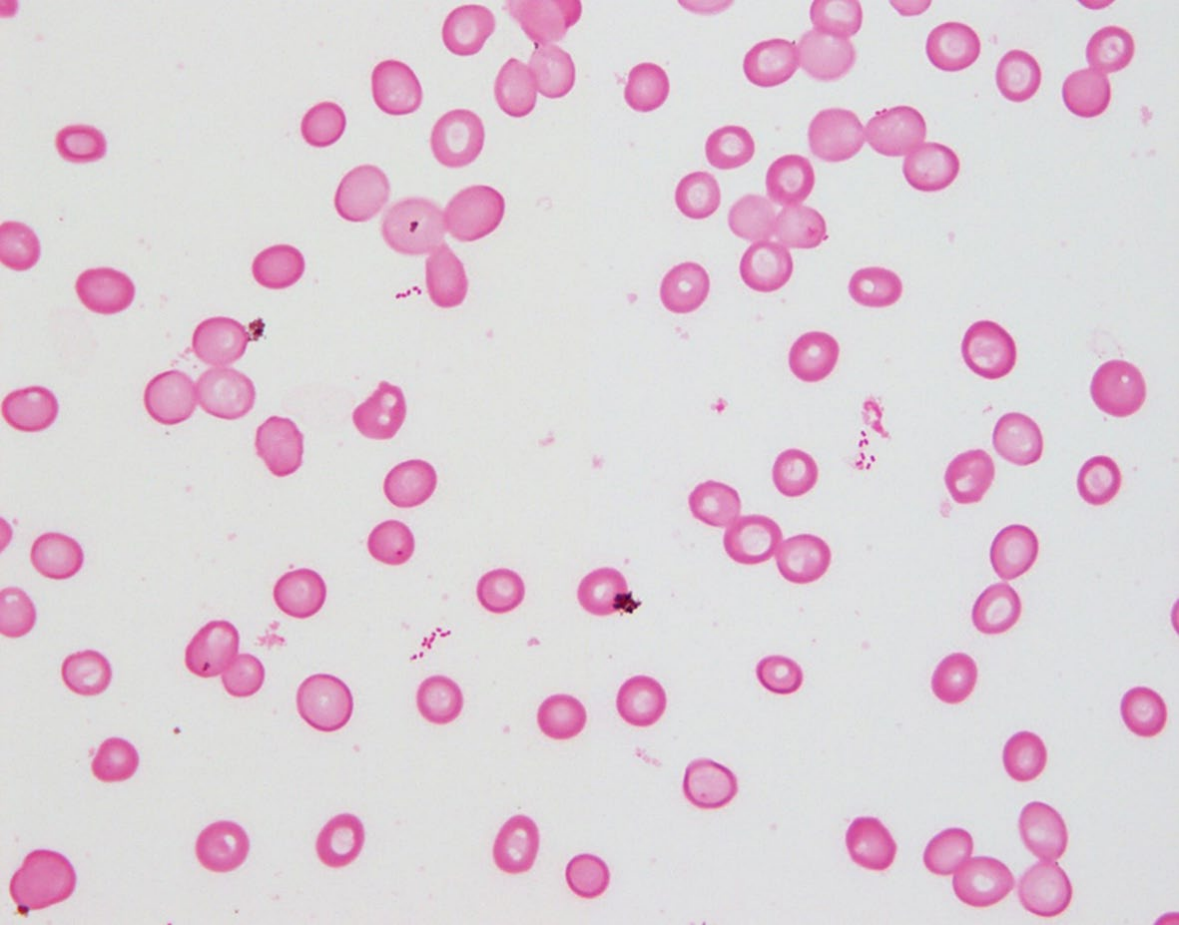
Gram staining of *Dialister micraerophilus* isolated from blood culture. Gram staining of blood culture that tested positive for *Dialister micraerophilus* shows small gram-negative bacilli. Magnification, × 1000 (oil)

Two anaerobic bottles of two sets of blood cultures were evaluated using the BACT/ALERT® VIRTUO® (bioMérieux, Inc., Marcy l’Étoile, France) blood culture detection system with BACT/ALERT® FA Plus and FN Plus bottles (bioMérieux, Inc.), which turned positive after 24 h 36 min and 37 h 54 min (Fig. 1). The two anerobic bottles were subcultured, as well as the pus sample, as previously described. The isolates were identified by using matrix-assisted laser desorption ionization time-of-flight mass spectrometry (MALDI-TOF MS) as previously described [17]. On the third day of incubation, tiny colonies of small gram-negative bacillus were observed on Brucella blood agar supplemented with hemin and vitamin K1 plates cultured under anaerobic conditions. *D. micraerophilus* was identified based on a score of 2.13 from one anaerobic bottle with an incubation period of 24 h 36 min. From the other anaerobic bottle with an incubation period of 37 h 54 min, *E. clostridioformis* and *E. lenta* were isolated and identified based on a high score ≥ 2.00. The subculture plates were incubated until day 5; however, no other species grew. Then, 16S rRNA gene sequencing was performed to identify *D. micraerophilus* isolates, as previously described [17]. This strain showed 100% (1440/1440 bp) similarity to *D. micraerophilus* DSM 19965 (accession number: AF473837). In addition, DNA was extracted from the pus sample using a MORA-EXTRACT DNA extraction kit (Kyokuto Pharmaceutical Industrial Co., Ltd., Tokyo, Japan). *D. micraerophilus* was detected in the pus sample by polymerase chain reaction (PCR) using *D. micraerophilus-*specific primers, dial micra_72F (5’-GGACATGAAAAGCTTGCTTT-3’) and dial micra_222R (5’-AGCGATAGCTTCTTCGATA-3’), and PCR conditions (20 s annealing at 57 ℃ and 20 s extension at 72 ℃) as previously described [14].

The minimum inhibitory concentrations (MICs) of various antimicrobial agents were determined via the broth microdilution method using IA40 MIC-i with Dry Plates Eiken (Eiken Chemical Co., Ltd, Tokyo, Japan) based on the Clinical and Laboratory Standards Institute (CLSI) standards [18]. The MICs were recorded after 48 h of incubation under anaerobic conditions as previously described at 35 ℃ (Table 1).

**Table 1:** MICs of the *Dialister micraerophilus* isolate

Antimicrobial agent	MIC (µg/mL)
Penicillin	≤ 0.06
Ampicillin	≤ 0.5
Ampicillin-sulbactam	≤ 0.5
Piperacillin-tazobactam	≤ 2
Ceftriaxone	≤ 1
Cefmetazole	≤ 1
Imipenem	≤ 0.25
Meropenem	≤ 0.25
Moxifloxacin	4
Clindamycin	1
Metronidazole	16

MIC, minimum inhibitory concentration

After diagnosing the patient with *D. micraerophilus* bacteremia, an intra-oral examination by a dentist revealed no sign of periodontal diseases or abscess. The infection resolved after drainage and empirical 7-day antimicrobial therapy with cefmetazole, followed by another 7-day oral amoxicillin-clavulanate treatment. The patient was discharged from the hospital on day 10. At the 1-week outpatient follow-up, the patient was well and without any complications.

## Discussion and conclusions

*D. micraerophilus* infection has rarely been described, and its clinical characteristics remain unclear. In this case, we diagnosed the patient with bacteremia caused by *D. micraerophilus, E. clostridioformis, and E. lenta*, associated with pyometra. A previous case report described a Bartholin’s abscess caused by *D. micraerophilus* [16]. In addition, *D. micraerophilus*, among *Dialister* spp., is mainly isolated from gynecological tract samples [1], although has been detected in vaginal samples [14, 15]. Therefore, *D. micraerophilus* may be associated with gynecological infections. No reported cases of bacteremia caused by *D. micraerophilus* exist in the available literature of case reports on bacteremia caused by *Dialister* spp. (Table 2), [5–8, 10, 19, 20, 21]. Although bacteremia caused by *D. pneumosintes* is mainly associated with dental infections or sinusitis [5–8, 19, 20, 21], a case of *D. pneumosintes* bacteremia associated with vaginosis has been reported [10], and *D. pneumosintes* has also been isolated from gynecological samples [1].

**Table 2:** Literature review on *Dialister* spp. bacteremia cases

No.	Reported year	Age (years)	Sex	No. of positive blood culture bottles for *Dialister* spp.	Isolated *Dialister* spp.	Identification method	Polymicrobial bacteremia (isolated organisms other than *Dialister* spp.)	Time-to-positivity of blood culture of *Dialister* spp.	Diagnosis (source of bacteremia)	Complicated with infections of oral cavity	Complicated with sinusitis	Antimicrobial treatment	Outcome	Reference
1	2002	17	M	One anaerobic bottle from two blood culture sets	*Dialister pneumosintes*	16 S rRNA gene sequence analysis	No	3 days (No detailed time was described)	Subdural empyema	No	Yes	Cefotaxime and metronidazole → oral amoxicillin and ofloxacin	Cured	[19]
2	2006	27	F	Unknown number of anaerobic bottles from three blood culture sets	*Dialister pneumosintes*	16 S rRNA gene sequence analysis	No	No detailed time was described	Postpartum vaginosis and pyogenic thrombosis of the ovarian veins	Not described	Not described	Imipinem and rifampicin	Cured	[10]
3	2015	62	F	Two anaerobic bottles from two blood culture sets	*Dialister pneumosintes*	16 S rRNA gene sequence analysis	No	36 and 41 h	Dental caries and sinusitis	Yes	Yes	Oral amoxicillin-clavulanate and ciprofloxacin → cefepime → cefepime and levofloxacin → levofloxacin	Cured	[8]
4	2016	78	F	One anaerobic bottle from two blood culture sets	*Dialister pneumosintes*	16 S rRNA gene sequence analysis	Yes (*Slackia exigua*)	30 h	Periapical abscess	Yes	Not described	Ceftriaxone and clindamycin	Cured	[5]
5	2021	13	F	One anaerobic bottle from unknown number of blood culture sets	*Dialister pneumosintes*	MALDI-TOF MS	No	34 h	Pneumonia	No	Yes	Ceftriaxon → piperacillin/tazobactam → meropenem → oral ciprofloxacin and sultamicillin	Cured	[6]
6	2021	30	F	One anaerobic bottle from unknown number of blood culture sets	*Dialister pneumosintes*	16 S rRNA gene sequence analysis	No	Detailed time not described	Neck and mediastinal abscess	Yes	Not described	Piperacillin/tazobactam → piperacillin/tazobactam and metronidazole → meropenem, vancomycin and oral fluconazole → oral amoxicillin/clavulanic acid and metronidazole	Cured	[7]
7	2022	73	F	Not described	*Dialister pneumosintes*	16 S rRNA gene sequence analysis	No	2 days (No detailed time was described)	Peritonsillar and retropharyngeal abscess	Yes	Not described	Ampicillin/sulbactam→ ampicillin/sulbactamand metronidazole → oral fluoroquinolone	Cured	[20]
8	2023	75	M	One anaerobic bottle from five blood culture sets	*Dialister pneumosintes*	MALDI-TOF MS	No	37 h	Aortic graft infection	No	Not described	Piperacillin/tazobactam and vancomycin→benzylpenicillin and gentamicin→oral amoxicillin/clavulanic acid	Cured	[21]
8	Present case	47	F	One anaerobic bottle from two blood culture sets	*Dialister micraerophilus*	16 S rRNA gene sequence analysis, MALDI-TOF MS	Yes (*Enterocloster clostridioformis* and *Eggerthella lenta*)	24 h 36 min	Pyometra	No	Not evaluated	Cefmetazole → oral amoxicillin/clavulanic acid	Cured	

F, female; M, male; MALDI-TOF MS, matrix-assisted laser desorption ionization time-of-flight mass spectrometry

In the present case, *D. micraerophilus* was not cultured from the drainage pus sample obtained from the pyometra uterus; this may have been due to the slow growth and tiny colonies of *D. micraerophilus*. However, *D. miraerophilus* was detected in the drainage pus sample by PCR using a specific primer. The patient had no other focus of bacteremia, including intra-oral infection, besides pyometra. Cases of bacteremia caused by *E. clostridioformis* or *E. lenta* in a patient with pyometra have been reported [22, 23].

In the present case, three anaerobes were isolated from blood cultures. Polymicrobial bacteremia caused by only obligate anaerobes is rare. The frequency of polymicrobial bacteremia cases implicating obligate anaerobes was reportedly 12.9–42.8% in cases of bacteremia implicating anaerobic bacteria (BIAB) [24, 25]. Dumont et al. reported that among 2,465 episodes of bacteremia including 144 BIAB episodes, polymicrobial bacteremia accounted for 301 episodes (12.2%), including 46 episodes involving at least one anaerobe (31.5% of all BIAB episodes) and 13 episodes involving only anaerobes (9.0% of all BIAB episodes) [24]. Watanabe et al. also reported that 42.8% (92/215 cases) of BIAB cases involved polymicrobial bacteremia, and 14.4% (31/215 cases) of BIAB cases were caused by multiple anaerobic bacteria [25]. In addition, Ransom and Burnham reported that among 158,710 blood culture bottles, 6,652 were positive anaerobic bottles, of which 384 (5.8%) contained 403 obligate anaerobes [26]. Moreover, 20.7% (81/392) of BIAB cases were polymicrobial cultures, including 73 cases with two species, 15 cases with three species, and 3 cases with more than three species. However, the frequency of polymicrobial bacteremia caused by only anaerobes was not described. In this study, blood cultures were performed using the BACT/ALERT® VIRTUO® system with BACT/ALERT® FA Plus and FN Plus bottles, similar to our study. Although polymicrobial bacteremia caused by three anaerobes is rare, *D. micraerophilus* was detected by PCR and *E. clostridioformis* and *E. lenta* was isolated from the drainage pus sample obtained from the pyometra uterus. Therefore, we finally diagnosed the patient with bacteremia caused by *D. micraerophilus, E. clostridioformis, and E. lenta* associated with pyometra.

*P. anaerobius* was isolated from the drainage pus sample, although *P. anaerobius* was not isolated from blood cultures in our case. Incubation of sub-culture plates continued until day 5. Cases of bacteremia caused by *P. anaerobius* have rarely been reported [27]. *P. anaerobius* was not detected using BACT/ALERT® FN Plus bottles or BD BACTEC™ Lytic bottles (Becton, Dickinson and Company, Franklin Lakes, NJ, USA) [28] in a previous study. The anticoagulant sodium polyanethol sulfonate inhibits *P. anaerobius* and was present in both bottle types, possibly explaining why *P. anaerobius* was not detected [27, 28]. A previous study showed that among 144 anaerobic bacteria isolated from blood cultures, 2.1% (*n* = 3) were *D. pneumosintes.* However, *P. anaerobius* was not detected [24].

The *D. micraerophilus* isolate in this case was identified by 16S rRNA gene sequencing and MALDI-TOF MS, as previously reported [16]; 16S rRNA gene sequencing [5, 7, 8, 10, 19, 20] and MALDI-TOF MS [6, 21] have also been used to identify *D. pneumosintes*.

Clinical breakpoints to interpret MICs do not exist for *Dialister* spp. The *D. micraerophilus* isolate showed MICs ≤ 0.06–1 µg/mL for β-lactam antimicrobial agents, 4 µg/mL for moxifloxacin, and 16 µg/mL for metronidazole. Although CLSI does not recommend that the broth microdilution method be performed to test for organisms other than *Bacteroides* spp. and *Parabacteroides* spp., the MICs for moxifloxacin and metronidazole in the *D. micraerophilus* isolate were high; moreover, Morio et al. reported a MIC_90_ of 8 for metronidazole in *D. micraerophilus* isolates as well as *D. pneumosintes* isolates [1]. Although antimicrobial susceptibility testing was performed using the Etest method, Cobo et al. reported that the *D. micraerophilus* isolate showed MICs of 12 µg/mL for metronidazole [16]. Morio et al. reported a MIC_90_ of 0.25 for moxifloxacin in *D. micraerophilus* isolates [1], which was lower compared with that of the *D. microaerophilus* isolated in our case.

In conclusion, we describe a case of a patient with pyometra, with bacteremia caused by *D. micraerophilus*, *C. clostridioforme*, and *E. lenta*. Thus, *D. micraerophilus* may be associated with gynecological infections. Clinicians should consider not only the oral site but also gynecological sites when searching to identify the focus of *D. micraerophilus* infection.

## Data Availability

The datasets used and analyzed during the current study are available from the corresponding author on reasonable request.

## Abbreviations

BIAB: bacteremia implicating anaerobic bacteria
CLSI: Clinical and Laboratory Standards Institute
MALDI-TOF MS: matrix-assisted laser desorption ionization time-of-flight mass spectrometry
MIC: minimum inhibitory concentration
PCR: polymerase chain reaction
